# Acute Decreases in Proteasome Pathway Activity after Inhalation of Fresh Diesel Exhaust or Secondary Organic Aerosol

**DOI:** 10.1289/ehp.1002784

**Published:** 2010-12-15

**Authors:** Howard M. Kipen, Sampada Gandhi, David Q. Rich, Pamela Ohman-Strickland, Robert Laumbach, Zhi-Hua Fan, Li Chen, Debra L. Laskin, Junfeng Zhang, Kiran Madura

**Affiliations:** 1 Environmental and Occupational Health Sciences Institute, Piscataway, New Jersey, USA; 2 Department of Environmental and Occupational Medicine, University of Medicine and Dentistry of New Jersey (UMDNJ)–Robert Wood Johnson Medical School, Piscataway, New Jersey, USA; 3 UMDNJ–School of Public Health, Piscataway, New Jersey, USA; 4 Department of Biochemistry, UMDNJ–Robert Wood Johnson Medical School, Piscataway, New Jersey, USA; 5 Rutgers, The State University of New Jersey, Piscataway, New Jersey, USA

**Keywords:** air pollution, diesel, inflammation, oxidative stress, proteasome, secondary organic aerosol, SOA, ubiquitin proteasome pathway, UPP

## Abstract

**Background:**

Epidemiologic studies consistently demonstrate an association between acute cardiopulmonary events and changes in air pollution; however, the mechanisms that underlie these associations are not completely understood. Oxidative stress and inflammation have been suggested to play a role in human responses to air pollution. The proteasome is an intracellular protein degradation system linked to both of these processes and may help mediate air pollution effects.

**Objectives:**

In these studies, we determined whether acute experimental exposure to two different aerosols altered white blood cell (WBC) or red blood cell (RBC) proteasome activity in human subjects. One aerosol was fresh diesel exhaust (DE), and the other freshly generated secondary organic aerosol (SOA).

**Methods:**

Thirty-eight healthy subjects underwent 2-hr resting inhalation exposures to DE and separate exposures to clean air (CA); 26 subjects were exposed to DE, CA, and SOA. CA responses were subtracted from DE or SOA responses, and mixed linear models with F-tests were used to test the effect of exposure to each aerosol on WBC and RBC proteasome activity.

**Results:**

WBC proteasome activity was reduced 8% (*p =* 0.04) after exposure to either DE or SOA and decreased by 11.5% (*p =* 0.03) when SOA was analyzed alone. RBCs showed similar 8–10% declines in proteasome activity (*p =* 0.05 for DE alone).

**Conclusions:**

Air pollution produces oxidative stress and inflammation in many experimental models, including humans. Two experimental aerosols caused rapid declines in proteasome activity in peripheral blood cells, supporting a key role for the proteasome in acute human responses to air pollution.

Epidemiologic studies have consistently documented increased risks of myocardial infarction and other ischemic events over the few hours to days after increases in ambient air pollutant concentrations, especially the particulate matter (PM) component (Peters et al. 2001, 2004; Pope et al. 2006; Rich et al. 2010; Zanobetti and Schwartz 2007). Increased risk of nonischemic cardiovascular (CV) events (ventricular arrhythmias, paroxysmal atrial fibrillation, heart failure) have also been reported (Rich et al. 2008; Wellenius et al. 2006). Several mechanisms by which PM may affect these diverse outcomes have been proposed, but informed understanding of these associations at physiological, cellular, and biochemical levels remains incomplete and lacks coherence across studies (Brook 2008; Scapellato and Lotti 2007).

Ambient PM is a complex mixture of particles with distinct physical and chemical characteristics that arise from various primary (hydrocarbon combustion) and secondary (photochemical smog) sources. Diesel exhaust (DE), a form of primary PM, can comprise up to 90% of urban PM (Salvi and Holgate 1999); secondary organic aerosols (SOAs) have been reported to constitute up to 50% of urban PM (Turpin and Huntzicker 1991; U.S. Environmental Protection Agency 1996). Whereas diesel particles contain insoluble elemental carbon cores, SOAs are composed of a water-soluble mixture of aldehydes, acids, and oxidants. In humans and animals, controlled exposures to freshly generated DE have been used to explore mechanistic pathways hypothesized to mediate acute CV responses to PM (Carlsten et al. 2007; Knuckles et al. 2008; Lucking et al. 2008; Mills et al. 2007; Peretz et al. 2007; Sunil et al. 2009; Törnqvist et al. 2007). Relatively few studies have examined the effects of SOAs on animals or humans. In rodents, age-dependent increases in pulmonary superoxide dismutase expression and nuclear factor κB activity were observed after SOA inhalation (Sunil et al. 2007). In contrast, no discernible nasal inflammation [white blood cells (WBCs), protein, interleukin (IL)-6, IL-8] or spirometry effects were observed in young healthy women (Fiedler et al. 2005; Laumbach et al. 2005).

Oxidative stress and inflammation have been proposed to underlie the observed associations between a diverse array of air pollutants and multiple cardiopulmonary effects (Dalle-Donne et al. 2006; Romieu et al. 2008). Closely linked to oxidative stress and inflammation is the proteasome pathway [ubiquitin proteasome pathway (UPP)], which mediates nonlysosomal proteolytic degradation of short-lived or oxidatively damaged intracellular proteins. The proteasome is a compositionally dynamic protease that performs approximately 90% of intracellular protein degradation. The well-characterized 26S proteasome contains an evolutionarily conserved catalytic particle (20S) attached to two regulatory (19S) particles. However, variable types of 20S complexes can associate with different regulatory particles, generating functionally distinct proteasomes such as the immunoproteasome (Glickman and Ciechanover 2002; Kloetzel 2004).

Initially considered only as an intracellular waste processor, the proteasome is now recognized as a key component in multiple regulatory pathways that employ signal-dependent protein degradation to control levels of diverse intracellular proteins. Substrates include proteins that are responsible for signal transduction and cell cycle regulation, as well as regulators of various inflammatory processes such as cytokine-induced gene expression and adaptation to oxidative stress (Obin et al. 1998). Increased proteasome activity has been noted *in vitro* after exposure to environmental stressors including oxidative damage and ultraviolet radiation, which suggests increased protein degradation (Dreger et al. 2009; Joseph et al. 2003; Seufert and Jentsch 1990). Pharmacologic proteasome inhibition leads to an accumulation of polyubiquitinated proteins, changes in cell morphology, increases in p53, nuclear factor erythroid 2-related factor 2 (Nrf2) dependent activation of antioxidant response elements, and apoptosis (Herrmann et al. 2010; Meiners et al. 2006; Meng et al.1999). Additionally, In a recent study, Salomons et al. (2009) demonstrated that proteasome activity is suppressed by exposure to heat shock, indicating that proteasome activity is sensitive to environmental stimuli. Dalle-Donne et al. (2006) showed that proteasome activity is also inhibited by oxidized (damaged) proteins that are resistant to degradation, which leads to metabolic or pathologic disruption of multiple cellular processes due to an accumulation of oxidatively damaged proteins.

We reported previously that the proteasome is involved in cardiac remodeling as an adaptation to afterload models of canine or murine heart failure (Depre et al. 2006). Rich et al. (2008) observed that increases in ambient PM aggravate chronic heart failure by acutely increasing right heart pressures in humans. These findings suggest a possible role for proteasome mediation of acute cardiac effects. It also raises the important question of whether oxidative stress in response to PM exposure alters proteasome activity and whether this contributes to acute PM-induced physiologic changes.

To begin to address these questions, we examined changes in proteasome activity in peripheral WBCs and red blood cells (RBCs) immediately after a 2-hr exposure of human subjects to freshly generated DE or SOA. Our findings that these exposures caused a significant decrease in proteasome activity suggests a novel biochemical pathway that may be important in the pathophysiologic response to air pollutants.

## Materials and Methods

### Subjects

Seventy-three men and women between the ages of 18 and 30 years were recruited from the Rutgers University community. Potential study subjects were screened for eligibility at the Clinical Center of the Environmental and Occupational Health Sciences Institute (EOHSI). Individuals with a history of treatment for CV disease, stroke, hypertension, or asthma, and those reporting a personal history of cigarette smoking within the previous 5 years were excluded. Because of simultaneous evaluation of DE and SOA effects on biomarkers of coagulation (manuscript in preparation), individuals were also excluded if they regularly used medications such as aspirin or drugs or supplements known to influence coagulation such as estrogen- containing contraceptives. After informed consent, but before study enrollment, subjects underwent a history and physical including electrocardiogram (ECG), spirometry, and standard laboratory evaluation. For subject comfort and safety, we excluded nine individuals who had clinically significant abnormalities, used an excluded medication, or had problems with phlebotomy (drawing blood). Before we initiated the exposures, an additional eight subjects dropped out of the study for personal reasons or because of scheduling problems. Subjects who met the inclusion criteria provided informed consent in compliance with the University of Medicine and Dentistry of New Jersey (UMDNJ)–Robert Wood Johnson Medical School institutional review board.

A total of 56 subjects were then scheduled for two or three 2-hr exposure sessions in random order: one to DE; one to clean air (CA) at least 1 week apart; and, when scheduling was feasible, to a third exposure to SOA. Both subjects and clinical staff were blinded to the exposure order. Of the 56 participants, 44 completed all three exposure sessions. Evaluation of the proteasome was added to the experimental design after initiating the primary study and required special processing of blood samples immediately after phlebotomy. Thus, of the 44 subjects enrolled in the primary study, 38 subjects who had samples available for assessing proteasome activity completed two exposures (DE and CA), and 26 participants completed all three exposures (CA, DE, and SOA). The reduced number of subjects who received the SOA exposure was due to logistical constraints in scheduling exposure sessions, as well as an *a priori* preference for assessing the effects of DE over the SOA when only two sessions could be scheduled for a subject.

### Experimental procedure

Subjects were instructed to fast after midnight before exposure sessions. At each exposure session (0800 hours), a technician verified subject adherence to the dietary, medication, and health status criteria. Blood was drawn for markers of platelet activation, nitric oxide metabolites, and proteasome activity. We performed noninvasive vascular studies (brachial artery ultrasound and EndoPAT) (Kuvin et al. 2003). Subjects then walked about 20 yards, took an elevator up two floors, and walked another 20 yards to the controlled environment facility (CEF) in the same building. After ECG (heart rate variability), the subjects were exposed for either 2 hr to DE [200 μg/m^3^ particulate matter ≤ 2.5 μm in aerodynamic diameter (PM_2.5_)], SOA (200 μg/m^3^), or CA (Table 1) while seated at a desk, usually reading or studying. The same battery of ECG, phlebotomy, and vascular measurements was repeated immediately after completing the exposure session (approximately 2–3 hr after baseline measurements).

### Proteasome activity analysis

Venous blood samples were collected in a cell preparation tube and transferred immediately to a nearby laboratory at room temperature. RBCs were separated from the buffy coat by dextran sedimentation. The red-cell pellet was resuspended in sterile phosphate buffered saline to a total volume of 12 mL, then centrifuged (2,000 × *g*) for 5 min at 4°C; RBCs were stored at −80°C. The buffy coat was suspended in IOTest 3 lysis solution (Beckman Coulter, Inc., Brea, CA) and centrifuged (2,000 × *g*) for 5 min at 4°C; supernatants containing plasma were then collected. The pellet was resuspended in IOTest 3 lysis solution and centrifuged again, and WBC pellets were collected and stored at −80°C for measurement of proteasome activity.

WBCs were suspended in 1-mL lysis buffer (50 mM HEPES, pH 7.5, 150 mM NaCl, 5 mM Na-EDTA, and 0.5% Triton X-100 protease inhibitor cocktail) and stored on ice. The cell suspension was transferred to a 1.5-mL microfuge tube and lysed five times with 10-sec pulses of a Sonic Dismembrator 100 (Fisher Scientific, Pittsburgh, PA), using a microprobe. Cell debris was removed after centrifugation at 4^°^C for 2 min (12,000 × *g*). Protein concentrations were determined using the Bradford assay (Bio-Rad, Bethesda, MD). Equal amounts of total protein extract (30 μg) were adjusted to 200-μL volume using proteasome assay buffer [25 mM HEPES, pH 7.5, 0.5 mM Na-EDTA, and 40 μM fluorogenic substrate *SUC*-Leu-Leu-Val-Tyr-AMC (LLVY-AMC; Boston Biochem, Cambridge, MA)]. Hydrolysis of LLVY-AMC requires the chymotryptic activity of the proteasome, which releases the highly fluorescent aminomethyl coumarin (AMC) moiety that is detected using Tecan Infinite F200 (TECAN–USA, Durham, NC) with the appropriate filter set. Proteasome activity measurements were performed in duplicate after 3 hr at 37^°^C. All measurements were generated in the presence and absence of the proteasome-specific inhibitor epoxomicin (200 ng/reaction) or dimethyl sulfoxide (DMSO, mock), to confirm the specificity of the assay. To measure epoxomicin-sensitive proteasome activity, the inhibitor was added to the protein extract 5 min before the addition of assay buffer containing LLVY-AMC to ensure complete inactivation of the proteasome. All results were adjusted to remove the nonspecific (epoxomicin-insensitive) values. Proteasome activity was measured as a unitless quantity separately for WBCs and RBCs.

### DE exposure session

All study exposures were conducted in the EOHSI CEF located on the Rutgers University campus. The CEF is a 25-m^3^ stainless steel–lined chamber in which air pollutant concentrations, temperature, and humidity are controlled. The temperature and relative humidity were 70°F and 35%, respectively, during all exposure sessions. DE was generated by a 5,500-W electricity generator (model YDG 5500EE; Yanmar America, Adairsville, GA) powered by a 406-cc displacement air-cooled engine. The engine was operated using number 2 undyed, low-sulfur, on-highway fuel. The engine is loaded with several space heaters and was maintained at 100% of rated capacity during each exposure session. The dilution and delivery system includes two single-stage mass reduction devices. The first-stage device is a 10-position butterfly valve that divided DE between the waste exhaust pipe and the second-stage mass reduction device, a variable-speed blower. After the two mass reductions, the desired amount of DE was introduced into the CEF air delivery stream to achieve the targeted DE PM concentration (200 μg/m^3^) within the CEF.

### SOA exposure session

The SOA generation system uses the gas-phase reactions between ozone and d-limonene in a large glass reactor. Spectrometry analyses have identified various products of reactions between ozone and terpenes (Fan et al. 2003). Generally, ozone attacks the > C = C < bond of the limonene and forms a primary ozonide, which rapidly decomposes to carbonyls and Criegee biradicals. Criegee biradicals react further to form compounds such as limonic acid, limonaldehyde, and limononic acid. These products have low vapor pressure and undergo gas-to-particle portioning, resulting in the formation of SOA. The SOA generated was carried by a heated CA stream at a flow rate of 10 L/min to the CEF, where residual ozone concentration was kept below 10 ppb.

### CA session

Ambient air was drawn into the CEF through a series of HEPA filters to remove particles and then further filtered through an activated carbon cartridge to remove gaseous impurities (e.g., volatile organics, ozone, oxides of nitrogen).

### Exposure system stability

All exposure systems were optimized to achieve stable concentrations in the CEF [Table 1 and Supplemental Material (doi:10.1289/ehp.1002784)]. Concentrations of PM_2.5_, nitrogen oxides (NO_x_), and carbon monoxide (CO) were monitored throughout each DE, SOA, or CA session. Real-time PM_2.5_ mass concentration was measured using a SidePak Aerosol Monitor (model 8520; TSI Inc., Shoreview, MN) calibrated with the same diluted DE or SOA in the CEF. Particle number concentration for the size range of 0.01–1 μm was measured with a condensation particle counter (Model 3007; TSI Inc.). NO_x_ were monitored using a chemiluminescent NO_x_ monitor (Thermo Electron Corp., Franklin, MA). CO was monitored using a Langan CO monitor (model T15v; Langan Products Inc, San Francisco, CA). Average concentrations for specific pollutants over the course of the study are shown in Table 1. The average total particle number concentration for SOA was 16,076 ± 5,233 particles/cm^3^, and the average mass was 193.8 ± 10.7 μg/m^3^. For DE, average values were 72,717 ± 3,605 particles/cm^3^ and 192.7 ± 7.2 μg/m^3^, whereas for CA they were 3,460 ± 1,963 particles/cm^3^ and 4.8 ± 3.9 μg/m^3^, respectively.

### Statistical analysis

For each subject visit, the preexposure proteasome activity, in WBCs or RBCs, was subtracted from the immediate postexposure activity values to estimate the change in UPP activity associated with a particular exposure session (CA, SOA, or DE). Next, the calculated CA proteasome activity change was subtracted from the calculated DE proteasome activity change, resulting in a grand delta for each subject for DE. This was repeated for SOA to generate a grand delta for each subject for SOA. This calculation provided a control for the effects of the experiment beyond the exposure. A mean grand delta for DE and a mean grand delta for SOA were then calculated to estimate an average change in proteasome activity associated with each exposure condition across all study subjects. Means and SDs were calculated to summarize the effects of exposure. Next, we used mixed linear models with *F*-tests and a random intercept to assess the effects of exposure (DE or SOA) on changes in proteasome activity after accounting for repeated measurements within subjects and sessions. At each stage of the analyses, we conducted diagnostics, including tests for normality and homogeneity of variances between groups. To determine if our results were sensitive to the different groups of study subjects included in the DE versus SOA analyses, we reevaluated a subset of 26 subjects for whom we had UPP activity data for all three exposures (DE, SOA, and CA). These analyses were performed on both WBCs and RBCs. Statistical significance was set at *p* < 0.05. Percentage estimates of effect are calculated based on the prevalues for the CA condition.

We used SAS/STAT software (version 9.1.3; SAS Institute Inc., Cary, NC) to perform all the analyses.

## Results

Table 2 shows the demographic characteristics of the 38 participants as well as the subset of 26 who received all three exposure conditions. All were healthy university students in their early to mid-20s, predominantly male (84%). Those who received the third (SOA) exposure session were of similar sex, race/ethnicity, and age distribution to the larger group. There were no significant differences in blood pressure, body mass index, or blood lipids between the groups. Table 3 shows the effects of exposure on WBC proteasome and RBC proteasome activity (mean ± SD) by exposure condition (DE, SOA, CA) and exposure status (pretest and posttest). In general, these normally distributed raw data show that postexposure values were lower for all three conditions for WBCs and for all but CA for RBCs.

Using mixed linear models to account for repeated measures within subjects and sessions, we analyzed changes in proteasome activity from preexposure to postexposure. Exposure to either DE or SOA produced a significant decrease in WBC proteasome activity when compared with CA [effect estimate = −1418.1 (−8.5%); 95% confidence interval (CI), −2811.3 to −24.8; *p* = 0.04] (Table 4). Analysis of the two pollutant aerosols separately showed SOA exposure was associated with a similar decline [effect estimate = −1928.5 (−11.6%); 95% CI, −3665.1 to −191.9; *p* = 0.03]; DE also decreased this activity, but did not reach statistical significance [effect estimate = −1070.9 (−6.4%); 95% CI, −2635.6 to 493.7; *p* = 0.17].

Analysis of RBC UPP was performed to determine if similar effects were observed as in WBCs. Using mixed linear models, borderline significant decreases in RBC proteasome activity were observed after exposure to DE or SOA, when compared with exposure to CA [effect estimate = −204.7 (−7.9%); 95% CI, −425.9 to 16.5; *p-*value = 0.06] (Table 4). Examining the effects of the two pollutant aerosols separately showed a decrease in RBC activity analogous to WBCs after DE exposure [effect estimate = −240.6 (−9.3%); 95% CI, −489.2 to 8.0; *p* = 0.05] or after SOA exposure [effect estimate = −151.8 (−5.9%); 95% CI, −428.4 to 124.9; *p* = 0.28] (Table 4).

## Discussion

In young, healthy human subjects, exposure to two distinct experimental aerosols (DE and SOA) produces rapid (within 30 min postexposure) and consistent decreases in proteasome activity in two different peripheral blood cell types. These results are highly novel and suggest that proteasome activity may be a useful early marker of physiologic response to PM. Although DE and SOA had similar PM mass concentrations, they were markedly different in mean particle number, chemical composition, and particle size distribution. The complexity of the aerosols makes it difficult to draw clear inferences about specific characteristics of the pollution mixture responsible for the effects on proteasome activity. However, the comparison with CA, as well as blinding of both subjects and investigators to the exposure conditions, essentially excludes psychological stress or other effects of study procedures as the explanation for decreased proteasome activity.

The mechanisms mediating decreases in proteasome activity after PM exposure are unknown. Oxidative and nitrosative stress have been directly associated with decreased proteasome activity, and they likely play a role in the response to PM air pollution (Bader and Grune 2006; Reinheckel et al. 1998). Both DE and SOA particles have been reported to induce an oxidative stress responses in animal and *in vitro* models (Miller et al. 2009; Sunil et al. 2007). In humans, induction of oxidative damage or an oxidative stress response has been repeatedly demonstrated in epidemiologic studies of PM exposure (Chuang et al. 2007; Romieu et al. 2008; Schwartz et al. 2005), with more limited findings after controlled PM exposures (Bräuner et al. 2007; Törnqvist et al. 2007). Xia et al. (2006) has proposed a hierarchical model of responses to PM in which increasing levels of oxidative stress transition from protective to proinflammatory and deleterious. Similarly, mild oxidative stress increases proteasome activity *in vitro* (Giulivi and Davies 1993; Reinheckel et al. 1998), which leads to downregulation of Nrf2-dependent antioxidant enzymes (Dreger et al. 2009; Herrmann et al. 2010), whereas higher levels of oxidative stress reduce protease activity and upregulate Nrf2. High levels of oxidative and nitrosative stress also severely damage proteins (and other macromolecules), which result in cross-linking, aggregation, and coagulation, that render them effectively resistant to proteolysis. From these data we cannot confidently infer whether our aerosol induces a mild or more substantial oxidative stress response, although the decreased proteasome activity suggests the latter. It is also possible that PM-induced decreases in proteasome activity are exacerbated by PM-mediated oxidative damage to mitochondria and consequent ATP depletion (Rinaldi et al. 2004; Shamoto-Nagai et al. 2003). Reduced ATP is associated with decreased protein translation fidelity, higher levels of proteasome-targeted nascent unfolded proteins, and impaired proteasome stability (Chuang et al. 2005).

As we did not add detergent to our proteasome activity assays, the major peptidase activity reflects the proficiency of the intact 26S proteasome, the complex that mediates the turnover of polyubiquitinated proteins, and is more susceptible to nitrosative damage (Grune et al. 2004; Reinheckel et al. 1998). These polyubiquitinated proteins include oxidatively damaged proteins known to interfere with proteasome function, either by exceeding its capacity or by poisoning its catalytic properties. Thus, our findings of decreased proteasome activity are consistent with and suggest significant levels of oxidative stress generated after inhalation of DE or SOA aerosols.

An increasing number of studies support the idea that PM alters vascular physiology (Mills et al. 2007; Peretz et al. 2007; Urch et al. 2005). Endothelial cells are protected *in vitro* against oxidative stress by pharmacologic proteasome inhibition (Herrmann et al. 2010; Meiners et al. 2006; Stangl et al. 2004). In cardiac myocytes, Nrf2-dependent transcriptional activation of antioxidative enzymes (e.g., superoxide dismutase and heme oxygenase) is also important for proteasome inhibitor-mediated protection against oxidative stress (Dreger et al. 2009). At present, it is unclear if the PM-induced proteasome inhibition is more of a response to oxidative damage or a direct inhibition of the enzyme system by air pollution or its oxidative consequences. Timing of outcome measurements is likely to be critical for a precise understanding of these pathways, as changes in proteasomal activity after mild *in vitro* oxidative stress were not evident until 1–4 hr after cessation of the stressor (Shang et al. 1997), and concepts of mild, high, and excessive oxidative stress need to be validated and experimentally modeled in humans. Overall, the effects of oxidative stress induced by SOA or DE are consistent with the reductions in proteasome activity that we have observed immediately (30 min) after fresh aerosol exposure.

An intriguing hypothesis to be addressed is that alterations in proteasome activity represent a fundamental pathway that interfaces between PM exposure, oxidative stress, and multiple physiological, vascular, and inflammatory effects in a complex web rather than a simple linear pathway. Proteasomes mediate the degradation of proteins that promote such divergent cellular outcomes as cell growth and apoptosis. Moreover, changes in global proteasome activity underlie multiple cell-survival mechanisms, some of which involve increased proteasome function, whereas others require reduced activity. Whether the role of UPP in air pollution responses is ultimately pathological, homeostatic, or merely a marker of effect remains to be elucidated. Further studies should refine oxidative stress and response outcomes in conjunction with more detailed subanalysis of proteasome function.

A methodological strength of our study was the double-blind, randomized cross-over protocol to compare the effects of controlled exposures with two separate aerosols and a CA control. However, several potential limitations should be considered when interpreting the data. The outcome data are limited to effects measured at a single time point. Although subjects were blinded to the identity of the exposures, DE and SOA both have mild characteristic odors at the concentrations used and thus could have been distinguished from CA but not readily from one another. However, previous experience with these exposures has not suggested a robust symptomatic response that might engender a generalized physiologic response (Fiedler et al. 2005; Laumbach et al. 2005).

It should also be noted that our proteasome activity measurements were limited to the mature 26S proteasomes. Any activity changes in the disassembled 20S subunit or the immunoproteasome, a distinct type of proteasome that participates in antigen presentation, would not be identified in our experiments. As other clinical studies demonstrate interactions between PM exposure and allergic responses, further study of the immunoproteasome is indicated (Gilliland et al. 2004).

The degree to which our findings of decreased proteasome activity in peripheral blood cells can be generalized to pulmonary, endothelial, and cardiac tissues awaits further investigation, but identification of the present systemic response suggests an even more robust pulmonary response. Although we did not directly assess links between oxidative stress and proteasome perturbation in this study, we did identify a biomarker of increased pulmonary oxidative stress, increased nitrite in exhaled breath condensate, in these subjects immediately after exposure to DE (Laumbach et al. 2010). Additionally, gene expression data from DE-exposed subjects showed increased expression of multiple oxidative stress response genes 24 hr after exposure (Pettit et al. 2010).

## Conclusions

Proteasome activity declined in healthy, young humans immediately after 2-hr inhalation of two distinct aerosol models of air pollution. Although our primary conclusion is that exposure to DE and SOAs elicits significant changes in proteasome function, presumably via induction of oxidative stress, further study is necessary to fully elucidate the mechanistic importance of these events. This will enable better understanding of the kinetics and effects of proteasome responses to air pollution.

## Figures and Tables

**Table 1:** Characteristics of three different exposures in relation to outdoor (background) concentrations.

Exposure/statistical results	Mass (μg/m^3^	No. of particles/cm^3^	Total hydrocarbons (ppm)		O_3_ (ppm)		CO (ppm)		NO (ppm)		NO_x_ (ppm)	
	Avg	Avg	Bkgrd	Avg	Bkgrd	Avg	Bkgrd	Avg	Bkgrd	Avg	Bkgrd	Avg
CA												
*n*	49	49	43	43	43	43	48	48	49	49	49	49
Mean	4.8 ± 3.9	3,460 ± 1,963	0.54 ± 0.15	0.71 ± 0.21	0.02 ± 0.00	0.02 ± 0.00	0.93 ± 0.37	0.93 ± 0.36	0.07 ± 0.32	0.06 ± 0.22	0.20 ± 1.27	0.19 ± 1.18
Max	21.3	8,549	0.87	1.17	0.02	0.03	1.99	1.96	2.04	1.45	8.93	8.32
Min	1.0	842	0.07	0.14	0.01	0.00	0.44	0.50	0.00	0.00	0.00	0.00

DE												
*n*	46	46	NA	NA	NA	NA	45	45	44	44	44	44
Mean	192.7 ± 7.2	72,717 ± 36,053	NA	NA	NA	NA	0.96 ± 0.40	4.49 ± 1.24	0.02 ± 0.02	3.76 ± 1.20	0.02 ± 0.02	3.89 ± 1.14
Max	206.3	137,524	NA	NA	NA	NA	2.25	6.89	0.08	6.67	0.10	6.80
Min	168.7	25,976	NA	NA	NA	NA	0.50	1.59	0.00	1.49	0.01	1.67

SOA												
*n*	43	43	43	43	43	43	NA	NA	NA	NA	NA	NA
Mean	193.8 ± 10.7	16,076 ± 5,233	0.56 ± 0.37	0.84 ± 0.34	0.02 ± 0.01	0.02 ± 0.00	NA	NA	NA	NA	NA	NA
Max	211.100	26,431	2.47	2.28	0.02	0.03	NA	NA	NA	NA	NA	NA
Min	148.600	6,865	0.04	0.36	0.00	0.01	NA	NA	NA	NA	NA	NA

Abbreviations: Avg, average; Bkgrd, background; Max, maximum; Min, minimum. NA indicates that a particular pollutant was not measured for a particular exposure condition. Mean values represent mean ± SD.

**Table 2:** Demographic and clinical characteristics of study participants without (*n* = 38) and with (*n* = 26) exposure to SOA.

Characteristic	*n* = 38	*n* = 26
Age	21.6 ± 3.3	22.2 ± 3.7
Sex [*n* (%)]		
Male	32 (84.2)	22 (84.6)
Female	6 (15.8)	4 (15.4)
Race/ethnicity^a^ [*n* (%)]		
White non-Hispanic	24 (64.9)	13 (52)
White Hispanic	4 (10.8)	3 (12)
Black non-Hispanic	1 (2.7)	1 (4)
Asian	8 (21.6)	8 (32)
Systolic blood pressure (mmHg)	114.7 ± 12.3	112.5 ± 11.7
Diastolic blood pressure (mmHg)	69.5 ± 9.1	69.9 ± 9.1
Body mass index (kg/m^2^)	24.6 ± 4.7	23.7 ± 3.6
Total cholesterol (mg/dL)	159.7 ± 33.7	162.7 ± 38.4
HDL cholesterol (mg/dL)	52.2 ± 13.7	51.5 ± 12.1
LDL cholesterol (mg/dL)	85.5 ± 28.9	90.0 ± 32.4
Triglycerides (mg/dL)	110.1 ± 58.6	105.5 ± 54.2

Abbreviations: HDL, high-density lipoprotein; LDL, low-density lipoprotein.

a Information on ethnicity was missing on one subject. Values represent mean ± SD unless otherwise specified.

**Table 3:** WBC and RBC proteasome activity measurements before and after each exposure.^a^

Exposure time point	WBC proteasome activity	RBC proteasome activity
Pre-DE	16755.8 ± 4354.2	2672.8 ± 1158.6
Post-DE	15068.3 ± 4632.3	2568.7 ± 1153.2
Pre-CA	16694.7 ± 5085.0	2587.4 ± 1197.4
Post-CA	16078.1 ± 5640.4	2723.8 ± 1075.6
Pre-SOA^b^	16719.9 ± 4668.5	2144.5 ± 1028.2
Post-SOA^b^	14173.2 ± 5850.3	2128.5 ± 979.9

Values represent mean ± SD.

a DE and CA data were available for 38 subjects.

b SOA data were available for 26 subjects only.

**Table 4:** Change in WBC and RBC proteasome activity associated with exposure to CA, DE, and/or SOA.^a^,^b^

Exposure	Change in UPP activity	95% CI	Individual *p*-value^c^	Overall model *p*-value^d^
WBC UPP activity				
Model 1				
Pollution (DE or SOA)	−1418.1	−2811.4 to −24.8	0.04	0.04
CA	Reference			
Model 2				
DE	−1071.0	−2635.7 to 493.8	0.17	0.08
SOA	−1928.6	−3665.2 to −192.0	0.03	
CA	Reference			

RBC UPP activity				
Model 1				
Pollution (DE or SOA)	−204.7	−426.0 to 16.5	0.06	0.06
CA	Reference			
Model 2				
DE	−240.6	−489.2 to 8.0	0.05	0.16
SOA	−151.8	−428.4 to 124.9	0.28	
CA	Reference			

a Model 1: a combined effect of DE and SOA on proteasome activity is estimated. Model 2: effects of DE and SOA on proteasome activity are estimated separately.

b *n* = 38 subjects.

c Obtained from solution for fixed effects separately for DE and SOA.

d Obtained from type 3 tests of fixed effects.
